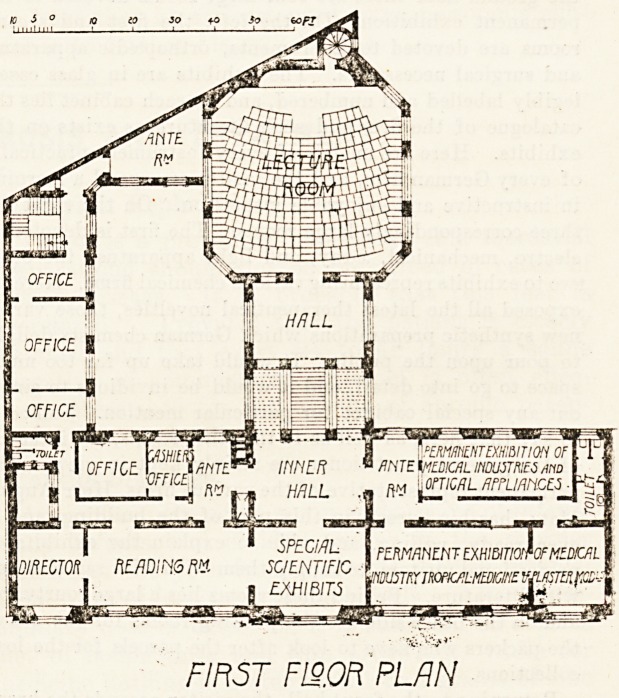# The Ideal Graduate Study Institution.—what Germany Has Done

**Published:** 1909-08-21

**Authors:** 


### Post-Graduate Medical Section.

### THE IDEAL GRADUATE STUDY INSTITUTION.—WHAT GERMANY HAS DONE.

### III.—THE KAISERIN FRIEDRICH HAUS.

"'Tis a house indeed, a very palace for our designs."
*Old Play.*

The Kaiserin Friedrich House stands on the Luisen platz, a quiet square just outside the rumble of Friedrichstrasse. Behind lies the maze of the Charité, a few hundred yards further on is the Augusta Hospital, close by are the Royal Clinics of Ziegelstrasse, and near at hand the Medical Jurisprudence Institution in Hannoverstrasse. Round the corner are the Veterinary College and the anatomical department, and equally close lie the various polyclinics. Trams pass the door bound for the large municipal hospitals, and the main stations for the overhead and the general railway lines are only a few minutes' walk away. A more central spot for the medical graduate it would be difficult to find in all Berlin.

A Description of the Building.

The accompanying elevation and ground plans give a good idea of the building as a whole, and it is only necessary to give a brief description of the main features. On the ground floor there are four large rooms devoted to the permanent exhibition. On the left, the first and second rooms are devoted to instruments, orthopædic apparatus, and surgical necessaries. The exhibits are in glass cases, legibly labelled and numbered, and on each cabinet lies the catalogue of the firm and such literature as exists on the exhibits. Here are models of every instrument practically of every German firm, and the visitor can spend a morning in instructive and enjoyable inspection. On the right are three correspondingly large rooms. The first is devoted to electro, mechanical, *x*-ray, and light apparatus; the other two to exhibits representing various chemical firms. Here are exposed all the latest therapeutical novelties, those varied new synthetic preparations which German chemists delight to pour upon the public. It would take up far too much space to go into detail, and it would be invidious to single out any special cabinet for particular mention. Suffice it to say that here, as well as in the other rooms, the interests of the general practitioner are mainly kept in view. The permanent representative of the various firms, Herr August Matz, has his bureau in this part of the building, and is ever ready, willing, and able to explain the exhibits to professional visitors, to supply them with trial samples and with literature. Behind these rooms lies a large courtyard, and on the other side are the packing rooms for the use of the packers who have to look after the parcels for the loan collections.

Returning to the front hall, the visitor ascends the broad flight of granite stairs and has an opportunity of admiring the simple yet effective entrance hall. On the first landing (Fig. II.) are the cloak-room, closets, and another large packing-room. On the first floor (Fig. III.), the ground plan of which is similar to that of the ground floor, there are on the left two large rooms again devoted to the permanent exhibition. In the one is gathered a large collection of optical instruments, microscopes, cameras, plates, etc. In the second is an even more interesting exhibition showing the merits of the various bathing and health resorts, the various "baths" and mineral springs. There are albums of views, plaster models, geological charts, analytical tables, pamphlets, details—everything the practitioner can wish to guide him in making a choice as to the suitability of this or that "kurort" for his patient. On the right is a large reading-room, well supplied with papers (unfortunately no English professional journal exchanges as yet with the institution, and in the permanent exhibition also one misses English firms, though some other foreign firms are represented). It is intended to establish in this reading-room a model general practitioner's library, containing copies of every book of interest to the practising physician, exclusive entirely of students' text-books. A beginning has already been made, and it is satisfactory to note that the large publishing firms are interesting themselves in the matter. The educative value of such a model library would be as great as that of the permanent exhibition. Behind the reading-room is the directors' room. From the reading-room one enters the main bureau, where cards for admission to the courses are given out and where the practitioner can obtain all the information he desires. The other bureau rooms, secretary's office, office of the official journal, and clerks' rooms are here.

Fig 1.—Front Elevation.

The Lecture Room.

Returning to the staircase one enters the large lecture-room or Hörsaal. This is a magnificent room, accommodating close upon 240 persons, excellently lighted and ventilated, and furnished with the best of modern contrivances. Thus the seats are of the well-known American "side table" model, the arm-rest serving as writing-desks, which can be raised or lowered at will. All the furnishing is of oak, and the seats are comfortable and broad. By pressing a button the lecturer can at will darken the whole room by means of electrically moved screens, which descend over the windows. Artificial lighting is provided for by a large number of centrally and side placed electric lamps. When all are lit the room is brilliantly lighted. On the lecture stage is a large plaster screen, over which a blackboard can be lowered for demonstration purposes. An up-to-date epidiaphanoscope, by means of which it is possible to throw on to the screen an enlargement of any ordinary picture or photograph, is here, the gift of the original makers, Carl Zeiss, Jena. This room is in constant use for courses, lectures, demonstrations, etc., and its acoustic properties are excellent, making it one of the finest lecture-rooms we have yet seen.

The Loan Collection.

On the third floor are the rooms for the State collection of specimens for demonstration purposes, which are loaned out to lecturers for demonstrations. The collection of wax models is one of the best in Europe and is daily being added to, the institution possessing its own modeller. Here is gathered together everything that one can desire for demonstration purposes, and space alone forbids us detailing the various excellent arrangements which clamour for the visitors' undivided attention at every step. The excellent collection illustrating the history of medicine, of which the nucleus was collected by Professor Hollander, is here, and the visitor can spend a day in viewing it. The collection of old Roman surgical implements, the armamentarium of the early German military surgeons, and the interesting collection of old prints and drawings, caricatures, and engravings of medical interest are particularly worth noticing. Of these we hope to give a fuller account in another article. Suffice it here to say that the institution is endeavouring to secure a complete collection of such interesting specimens, and to take photographs and make lantern-slides of all these exhibits for the benefit of lecturers who wish to demonstrate on medical history. A fairly complete catalogue has already been printed, and several lots of diapositives are available for loan purposes. Equally interesting, and of perhaps greater educative and practical value, is the collection of exhibits illustrating the nursing of the sick pathological and microscopical work, and is arranged for a membership of twenty in each course. Each member of the course has his own bench, with revolving seat, drawers, and slide cabinet.

The lamps used are not the ordinary electric light which is particularly trying to the eyes, but a special model, designed by Dr. Lowin, and specially made for the institution. In this model gas mantles are used, and the light given is steady, clear, and very well suited for microscopic work. Here, too, is a projection apparatus for demonstration purposes, and an arrangement for automatically darkening the room, similar to that which exists in the large Hörsaal. As an illustration of the thoroughness with which the institution adheres to its ideal to make the courses free to practitioners, it may be mentioned that every member is supplied free of charge with a complete microscopic outfit, Zeiss instrument, with two ordinary and an oil immersion objective, slides, staining reagents, and all necessary addenda. These outfits, of course, belong to the institute, and are returned when the course is completed. In the adjoining laboratory for bacteriological work, the arrangements are equally thorough. One need only mention the fact that no taps are provided on the benches (to obviate the injudicious washing of slides

KAISERIN FRIEDRICH HAUS

GROUND FLOOR PLAN·

FIRST FLOOR PLAN

In this there are models of bedsteads and bedding, night lights, arrangements for saving labour in wards, for sick-room cookery, for general nursing and for ambulance work. These models, like all the rest of the exhibits in the State collection, are also available for loan purposes and are frequently used for demonstrations at branch centres.

### The Laboratories.

On the top floor are the working rooms, in which practical courses are held, and here one is at once struck with the thoroughness with which every detail has been considered, and with the excellent results that have been obtained. The lavatory accommodation is thoroughly satisfactory and efficient, and easy access is obtained to the rooms by means of the automatic electric lift with which the building is provided. There are two large laboratories, in each of which courses are held daily at times to suit the members, who are all general practitioners engaged in practice in Berlin or its suburbs. The first laboratory is used for and the escape of culture media into the general drains). Here is found a specially-constructed electrical centrifuge with glycerine speed indicator. Attached are the demontrator's private room; then come a washing room, an incubation room, a room for special experimental work, the workshop of the waxwork modeller, and the lavatories. On the other side is a large Röntgen-ray room, replete with all the latest apparatus, and with a small developing and dark room attached. Courses are given here, and are very popular. The outfit is one of the best in Berlin, and the photographs obtained, both by the direct and by the "relief" methods are excellent. Behind this is a large, airy, well-lighted photographic studio, with apparatus for taking cinematographic films. Attached to it is a copying room, and a dark room with an automatic lighting arrangement by means of which the operator can obtain red, yellow, green, violet, or white light at will. A large amount of work is done here, especially in cinematographic photography, for the value of the cinematograph for demonstration purposes is fully recognised by the Central Committee, and both films and instruments are frequently loaned to branch centres. As an example of the value of such demonstrations, the recent films showing the gait in various forms of spastic paralysis may be mentioned.

This rapid outline of the Kaiserin Friedrich Haus will suffice to give the reader a general idea of the institution. What it cannot give is the impression which a personal inspection of the whole makes on the visitor. There is nothing cheap or shoddy, for it is recognised that in such things true economy lies in purchasing the best apparatus and the most durable materials. From an architectural point of view the building is almost ideal; from the demonstrator's and student's point of view it is really ideal. It is a pleasure to work in these laboratories, as it is an enjoyment to spend a morning in the exhibition rooms. Anyone, in fact, who desires a practical illustration of the ideal graduate place of study, should visit this institution and spend a day in becoming acquainted with the system on which it is worked and the practical usefulness of its methods. He will find no more courteous cicerone than the director, and will return from his visit, as we returned, with a feeling that here at least is something which is worth imitating on this side of the Channel.

## Figures and Tables

**Fig. 1. f1:**
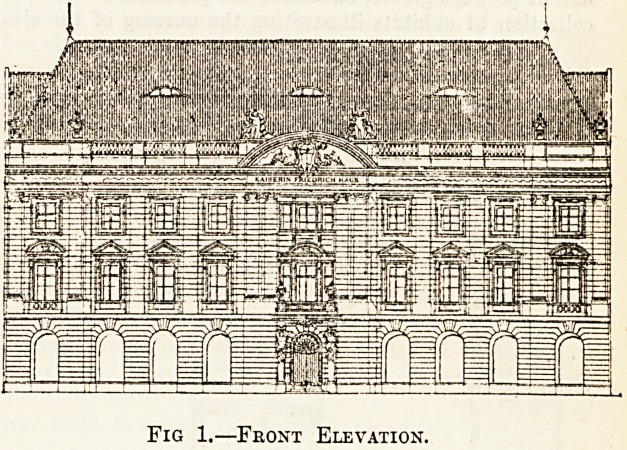


**Figure f2:**
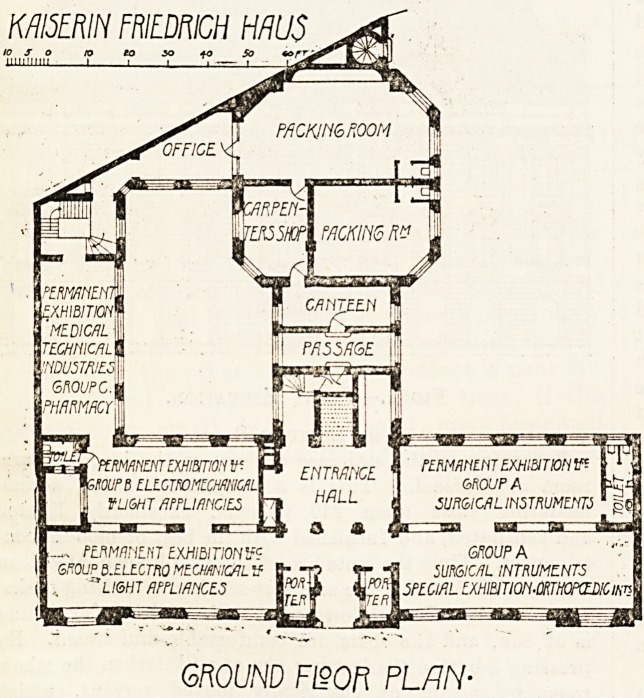


**Figure f3:**